# Development of a multidimensional military readiness assessment

**DOI:** 10.3389/fresc.2024.1345505

**Published:** 2024-03-20

**Authors:** Riley C. Sheehan, Michael Vernon

**Affiliations:** ^1^ Henry M. Jackson Foundation for the Advancement of Military Medicine, Inc., Bethesda, MD, United States; ^2^ Center for the Intrepid-Brooke Army Medical Center, Fort Sam, Houston, TX, United States; ^3^ Department of Physical Medicine and Rehabilitation, Uniformed Services University of the Health Sciences, Bethesda, MD, United States

**Keywords:** return to duty, virtual reality, simulation, cognitive, physical

## Abstract

There is a need to be able to accurately evaluate whether an injured service member is able to return to duty. An effective assessment would challenge and measures physical and cognitive performance in a military-relevant context. Current assessments are lacking in one or more of these aspects. The simulation and data capture abilities of virtual reality systems are promising for use as the basis of multidimensional assessments. The team has previously developed a military-specific assessment in the Computer Assisted Rehabilitation Environment (CAREN) called the Readiness Evaluation During simulated Dismounted Operations (REDOp). Due to notable limitations in the original assessment, we have developed the next iteration, REDOp2. The assessment is able to challenge and measure a broader range of physical and cognitive performance domains in a more streamlined fashion. While limited to facilities with a CAREN, REDOp2 has the potential to provide an effective tool for highly trained and experienced wounded service members that require thorough assessment prior to returning to duty to ensure the safety of the team and mission. This methods paper describes the specific limitations in REDOp, how they were addressed in REDOp2, and suggested next steps to prepare the assessment for implementation.

## 1 Introduction

Readiness and world-wide deployability are top priorities for the U.S. Armed Forces. Unfortunately, injuries and illness often limit service members (SM) from being able to fully perform their military duties which subsequently reduces military readiness. In particular, musculoskeletal (MSK) injuries have a significant negative impact on military readiness, constitute a high proportion of theater evacuations (1, 2), and are responsible for the greatest consumption of Military Health System resources (3). Mission performance and readiness are also affected by cumulative non-traumatic injury caused by the rigorous demands of military training and deployment. These non-combat MSK injuries accounted for 48% of the medical evacuations from an Army Brigade Combat Team during the war in Iraq (4, 5). Overall, MSK injuries impair the ability of SMs to effectively perform the highly demanding tasks of military duty and are, not surprisingly, responsible for the greatest percentage of limited duty days (6). While MSK injury has the greatest impact on readiness, it primarily affects physical readiness. There are many other injuries/diseases that can impair other factors of military readiness, such as traumatic brain injury causing problems with memory, reaction time, and concentration degrading mental readiness (7, 8).

The military expends a significant amount of resources to recruit, train and outfit a SM to be ready to deploy (9). Consequently, each SM represents a significant investment to the Department of Defense and the nation. Military service, especially in a deployed environment, places high physical and mental demands on SMs requiring them to function at their peak capabilities in order to assure mission success. Success on the battlefield is also enhanced by having SMs with past experience, especially in combat. This experience can be invaluable to a unit's safety and success during subsequent deployments. Therefore, getting highly trained and experienced wounded service members “back in the fight” after injury is extremely valuable, not only to unit success, but in building morale and unit cohesion. At the same time, caution must be taken before returning an injured service member back to duty, as their risk of re-injury or not being able to fully function would ultimately jeopardize the effectiveness and safety of the unit. Thus, it is imperative that there is an effective measure of military readiness and that the return to duty (RTD) decision is as informed as possible.

Despite the importance and potential ramifications of the RTD decision, there are no objective, multidimensional, and validated measures that are used consistently to inform RTD decision-making. The decision is made primarily with the information at hand which often relies heavily on subjective feedback from the SM's care team and chain of command and, potentially, any performance measures that may have been collected as part of his/her care. Therefore, objective, evidence-based assessments that inform RTD decisions are necessary to maximize readiness.

In order to effectively develop tools to predict successful RTD following injury, there needs to be an effective measure of military performance. Military service places high demands across a range of physical, cognitive, and emotional domains. SMs must be able to make rapid, accurate decisions while performing physically demanding tasks in a highly distracting and stressful environment. While there are individual assessments that are administered at different points during military service, there are no established comprehensive assessments of military performance across the various disciplines. Thus, it is difficult to effectively assess and quantify the multifaceted nature of military service. Further, there is no quantitative measure to serve as a criterion to develop and validate these readiness assessments.

Current evaluations have significant limitations as they have yet to assess the multifaceted demands of military service in a manner that is clearly militarily relevant. Many of the standard clinical measures lack face validity with SMs as it can be difficult to interpret how results on these assessments relates to performance on military occupational tasks. Additionally, several specifically designed assessments focus solely on symptom identification and lack face validity and relevance to military occupational tasks (10). The Army Combat Fitness Test (11) and Occupational Performance Assessment Test (12–14) were developed to evaluate whether SMs have the physical abilities, or potential, to perform their military duty. However, these assessments only focus on the physical aspect of military tasks and were developed for healthy SMs, thus lacking the ability to assess the multifaceted demands of military service in an injured population in a RTD context. Other assessments are overly specialized for a specific diagnosis or symptomology (7, 8, 15–19). For example, the Assessment of Military Multitasking Performance (7, 8, 16–19) and subsequent Portable Warrior Test of Tactical Agility (20, 21) focus on evaluating SMs with traumatic brain injury, but are not clearly relevant and appropriate for SMs with other diagnoses such as MSK injury. There are also assessments like the Military Functional Assessment Program (22, 23) that are highly time and resource intensive being administered over 5 days using specialized, high-tech equipment found only at large military installations, which limits feasibility for individual patient use. Thus, there is an unmet need for multidimensional readiness assessments.

The primary limitations of readiness assessments involve the difficulty of challenging and evaluating multiple domains (e.g., physical and cognitive) within a military relevant context. The use of virtual reality has the potential to address these limitations. The combination of the programmability and flexibility in designing and developing the virtual environment allows for the creation of complex challenges within military relevant contexts, all with integrated data capture. While a number of different virtual reality and simulation systems have been used for military training and mission rehearsal (24), this technology has not been leveraged for the assessment of SM readiness.

Responding to the need for objective, comprehensive assessments of military readiness, our group at the Center for the Intrepid (CFI) has developed a number of tools to address the shortcomings of current assessments listed above. We created the Readiness Evaluation During simulated Dismounted Operations (REDOp) assessment leveraging the simulation capabilities of the Computer Assisted Rehabilitation Environment (CAREN). The REDOp consists of a simulated dismounted patrol over variable terrain with periodic “ambushes” where numbered targets appear, and participants have to make rapid shoot/don't-shoot decisions. The assessment was able to identify key deficits and performance-limiting factors in SMs with lower extremity trauma and amputation while completing a simulated dismounted patrol. The assessment demonstrated excellent reliability (intraclass correlation coefficient (ICC) > 0.78) for key embedded performance measures quantifying activity tolerance, stability, and shooting performance in both able-bodied and injured SMs (25). The REDOp assessment is currently being used by clinicians at the CFI to evaluate patient rehabilitation progress and identify deficits in response to the simulated patrol.

Despite its demonstrated utility, the REDOp assessment has key deficits that limit its effectiveness as a readiness assessment and hamper widespread adoption (Table 1). Through discussions with participants, clinicians, and stakeholders, the research team has identified six primary deficits in the original REDOp assessment.

(1) Limited assessment of the cognitive domain: The only cognitive assessment in REDOp is the shoot/don't shoot aspect of the ambush task. To be a truly multidimensional assessment, it must better assess the cognitive domain to include object identification and memory as well as rapid decision making.
(2) Ambush task did not provide sufficient challenge: The ambush task consisted of waves of targets appearing and friend/foe targets determined based on even/odd number. Participants did not have a problem determining odd or even and used the maximum amount of time to clear the targets. To be able to effectively evaluate military-relevant rapid decision-making abilities, there needs to be a more complex determination of friend or foe and an imposed constraint to ensure decisions are made as rapidly as possible.
(3) No measure for aim stability: Participants with lower limb and back injuries often have difficulty decoupling upper and lower body movements. As a result, they can struggle with maintaining a stable shooting platform while walking. As the ability to maintain effective aim while moving is critical for many engagements, it is important to be able to evaluate this in a readiness assessment.
(4) The speed and movements of the platform were not realistic and relevant: The treadmill speeds and angles were modeled after a common treadmill stress test where the speed and incline progressively increase. While this is effective for exercise testing, it does not emulate what a SM would experience on a real mission. It is important for a military assessment to simulate military challenges to elicit more realistic responses and performance.
(5) Difficulty comparing performance between participants: The assessment was based around recurring blocks and the participants completed as much as they could. As such, different participants would have a different number of measures depending on how many blocks they completed. This made it difficult to compare the relative performance of participants. For REDOp, only the measures during the first block were used which did not provide the full picture of their performance. An effective assessment should enable comparison regardless of how they performed.
(6) Data collection and processing was resource intensive: The assessment required the accurate placement of 57 markers on the participant to track their whole-body movement. This required specially trained staff to perform and led to long participant setup times. That full-body marker data also had to be cleaned and processed after the collection again requiring trained personnel and extensive time. As a result, it was time and resource intensive to complete the assessment and there was a notable delay from the completion of the assessment to when a report could be generated. To maximize utility, the assessment should be able to be administered with minimal training and it should be able to generate a report soon after the completion of the assessment.

**Table 1:** Description of the primary limitations in the original REDOp, how they were addressed in REDOp2, and associated metrics with the solution.

REDOp limitations	REDOp2 solutions	Metrics
Limited assessment of the cognitive domain	Identification and Recall Task, Improved Ambush shooting task	Recall error rate, Accuracy, Precision, Reaction Time
Ambush task did not provide sufficient challenge	Increased shoot/don’t shoot decision difficulty with symbol matching task and reduced decision time to 3 s	Accuracy, Precision, Reaction Time
No measure for aim stability	Added weapon tracking task to evaluate ability to decouple upper and lower body to maintain stable aim while walking	Percentage of time crosshairs on target
Speed and movements of the platform were not realistic and relevant	Platform tracks the terrain while participants walk at forced march pace (1.6 m/s) with periodic 3–5 s rushes	
Difficulty comparing performance between participants	Used exponential weighted average approach to calculate overall scores regardless of the number of blocks completed	Overall scores
Resource intensive data collection and processing	Reduced marker set to 3 markers, consolidated data capture to one system, and created custom data processing program to process data and generate report	Able to export and process data and generate a report within 10 min of participant completing the assessment

We have used the experience gained from the development of REDOp and other military scenarios, and through researching other RTD and readiness assessment efforts, to address these limitations and develop an updated multidimensional military assessment, REDOp2. The purpose of this methods paper is to outline the key revisions and features of the newly developed REDOp2 for the assessment of return-to-duty potential in key military personnel. Additionally, the authors outline the next steps needed to validate the assessment and move it closer to implementation.

## 2 Materials and equipment

**Figure 1:**
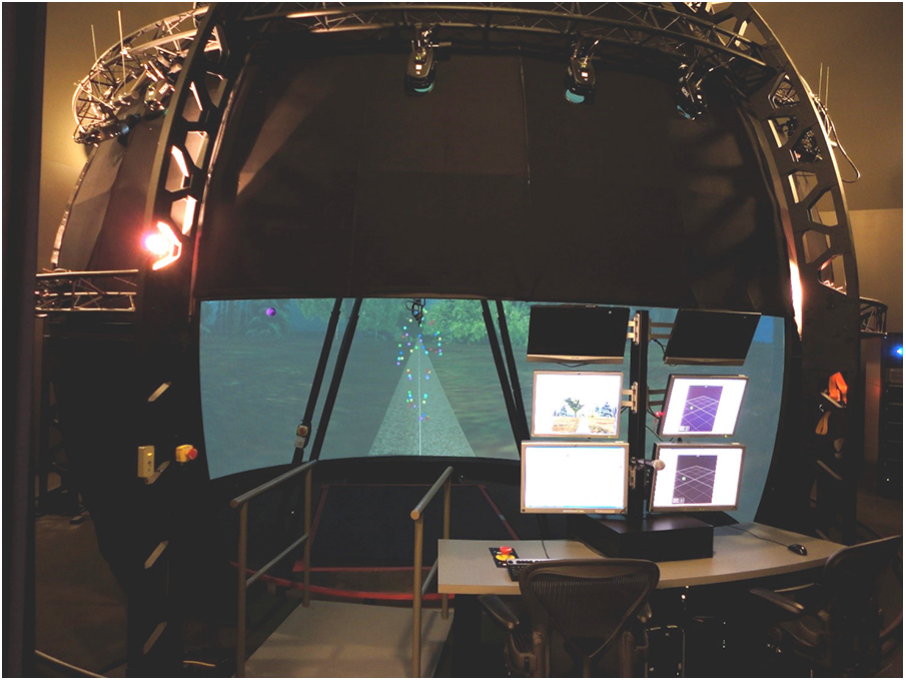
A photo of the computer assisted rehabilitation environment (CAREN). The system is able to simulate a wide variety of tasks and scenarios.

Similar to previous projects, we leveraged the simulation capabilities of the CAREN (Motekforce Link, Amsterdam, Netherlands). The CAREN system consists of a 300 deg dome screen around a 6 degree-of-freedom motion platform with embedded 2 m × 3 m treadmill (Figure 1). The system has an integrated 30-camera motion capture system (Vicon Motion Systems, Oxford, UK) to allow data capture and interaction with the virtual environment. The integration of all of these components allows for the platform to match the virtual terrain, which, combined with the 300 deg projection, provides a truly immersive experience for the participant.

The scenario also utilizes a simulated M4 rifle. The trigger pulls are transmitted wirelessly to the computer and communicates through standard game controller protocols. Markers on the weapon are used to track the location and define a pointer displayed as virtual crosshairs on the screen and used for the aiming and interacting with the scenario. Heartrate was captured using a chest-worn heart rate monitor (Polar Electro, Inc., Bethpage, NY).

## 3 Methods

### 3.1 Assessment components

In the planning of the development for REDOp2, we took a number of steps to ensure that the assessment would assess military-relevant tasks and quantify key performance domains. We used the Functional Activities in the DA Form 3,349 Physical Profile and the Soldier's Manual of Common Tasks to identify key activities to base the assessment components around. In addition to drawing on feedback from patients who had gone through previous CAREN scenarios, we elicited input from an advisory panel of key stakeholders (e.g., clinicians, special operators, Physical Evaluation Board members) about what domains to evaluate. As with the original REDOp, we decided to base the scenario around a simulated dismounted patrol. Participants attempt to complete a 4 km (∼2.5 mile) patrol over variable terrain at forced march pace of 1.6 m/s. The patrol is broken up into 400 m blocks during which the participant performs an identification and recall task followed by a 3–5 s rush (getting up to ∼3 m/s) before slowing down to a walk (0.4 m/s) and completing either a weapon tracking task or an ambush shooting task. At the end of each block, participants report on the identification and recall task as well as their rating of perceived exertion and pain. Participants go as long as they can until they either complete the whole 4 km patrol, ask to stop, or are stopped by the team for safety reasons. Throughout the assessment, we capture the motion of markers on the feet and lumbar spine and monitor heart rate using a chest strap mounted sensor.

**Figure 2:**
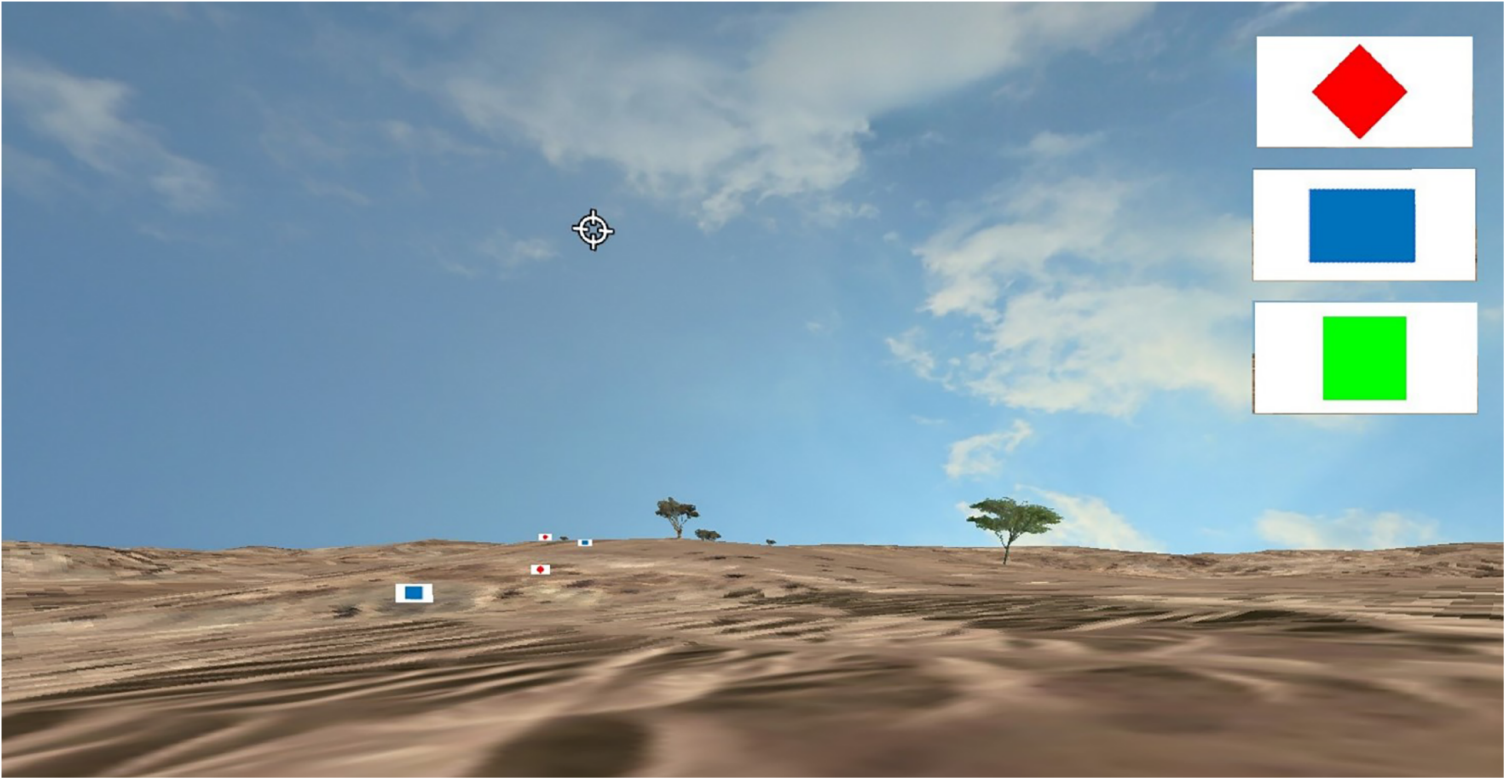
Example of the identification and recall task where the 3 map symbols on the right are distributed across the landscape and must be identified over the 400 m block and the number of each type recalled at the end of the block.

*Identification and Recall*: For each block, 5–15 map symbols are placed across the terrain. These symbols include red diamond (Hostile), blue rectangle (Friendly), and green square (Neutral). Participants need to identify and remember the number of each type of symbol they encounter and report those numbers at the end of the block (Figure 2). The task is meant to assess symbol identification and recall with distractions.

**Figure 3:**
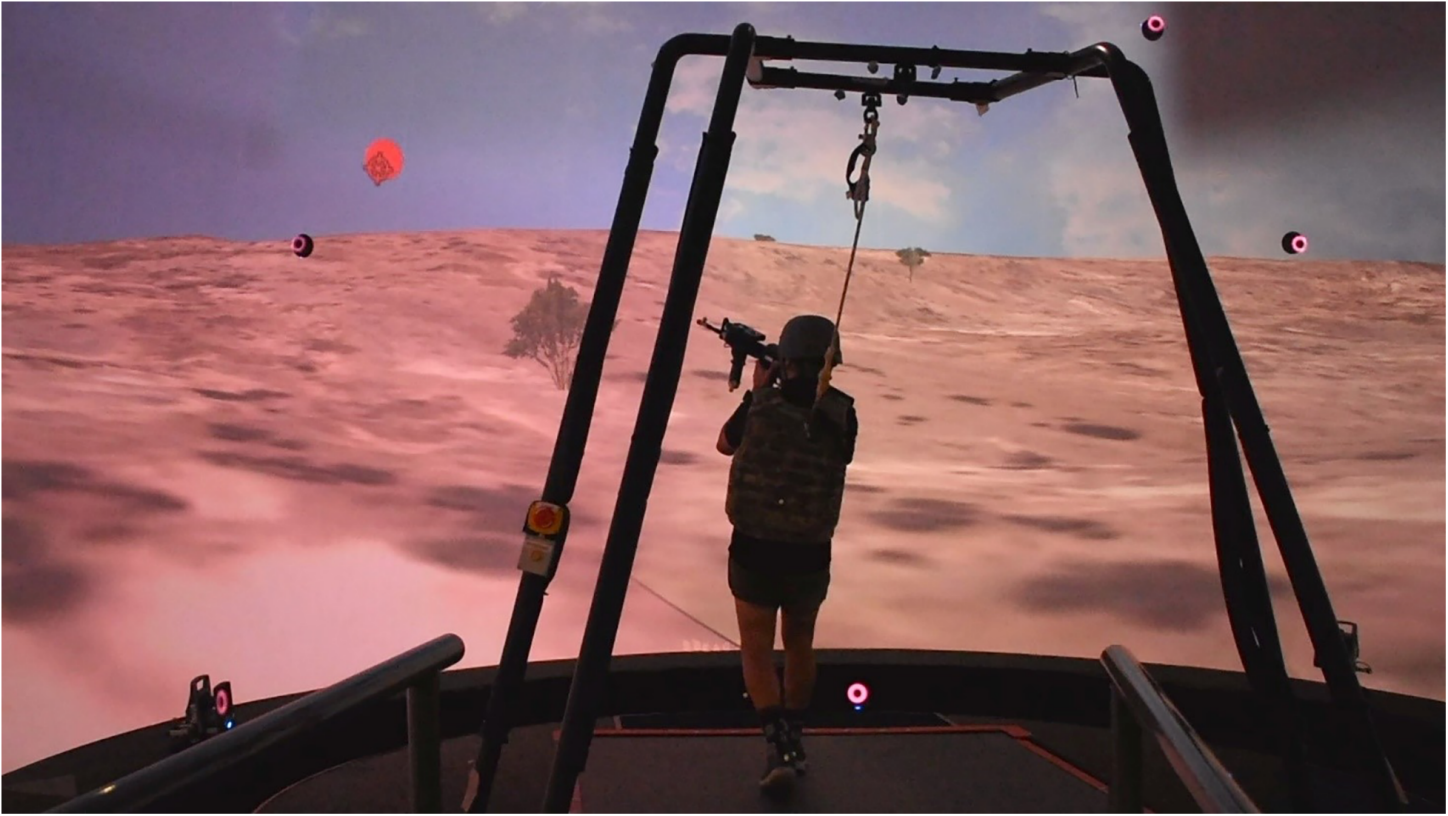
A participant completing the tracking task. They must maintain the crosshairs over the circle target while walking.

*Weapon Tracking*: While walking at 0.4 m/s, participants must place the virtual crosshairs over a target (white circle) and keep them on the target as consistently as possible for 10 s (Figure 3, Supplementary Material—Weapon Tracking Video). The task includes 5 target locations presented one after the other. The locations include 3 static locations (Left, Center, Right) and 2 moving targets (from left and from right) where the target makes a sinusoidal path across the screen. The task is meant to assess the ability to decouple upper and lower body movements and maintain a stable aim while walking.

**Figure 4:**
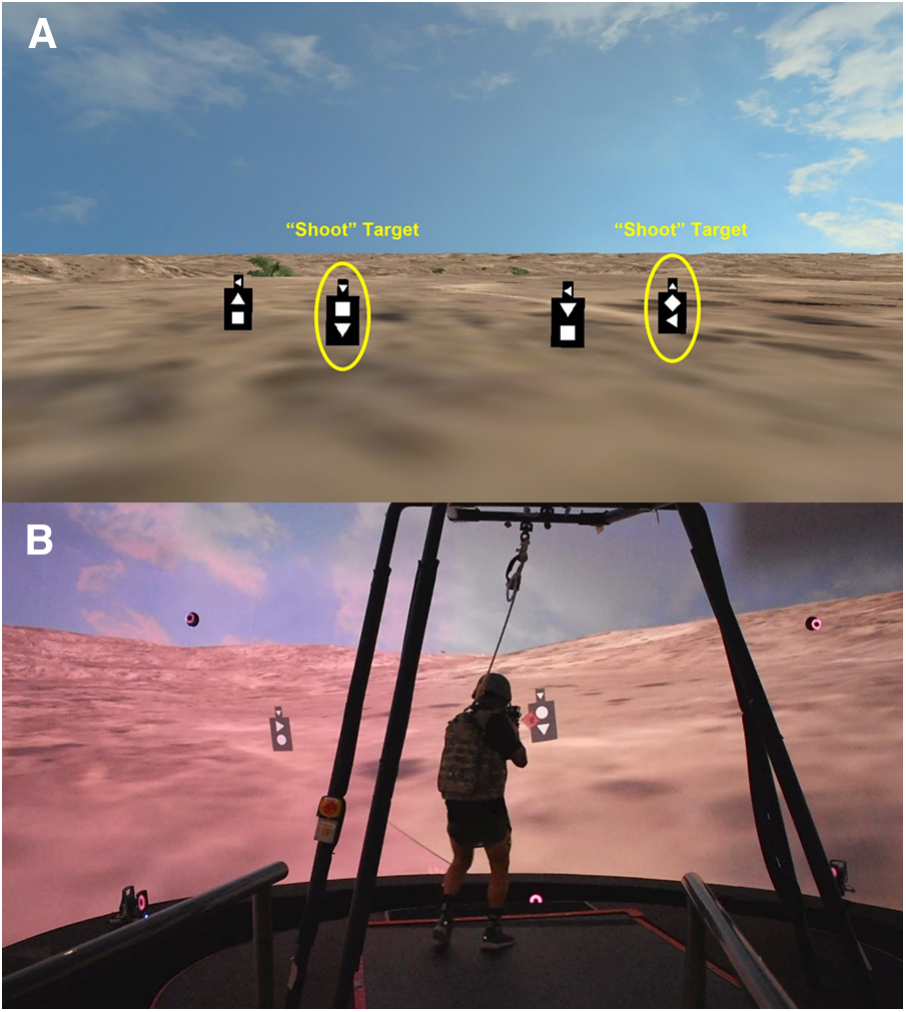
(**A**) Example of the targets and the symbol matching scheme for the ambush task. (**B**) Participant completing the ambush shooting task.

*Ambush Task*: 10 waves of 1 or 2 targets (5 waves each) are shown for 3 s. The targets have 3 shapes on them, one on the head and 2 on the body. If the shape on the head matches a shape on the body or there is a diamond on the body, that target is considered a “shoot” target (Figure 4, Supplementary Material—Ambush Task Video). Participants must make a rapid decision and shoot the “shoot” target before it disappears. The presentation of the targets is different for every block. The task is meant to assess reaction time and rapid decision making.

### 3.2 Assessment measures

The assessment includes a variety of embedded measures to evaluate domains including endurance and activity tolerance, gait stability, identification and recall, motor control, reaction time, and decision making (Table 1). Many variables are captured in each block. By repeating the different tasks over the course of the patrol we are able to look at the change in performance over time and identify relationships between cognitive function and physical exertion. An exponentially weighted average is used to combine the repeated block variables into an overall assessment score for the repeated variables. The weighting for each block is calculated as $\alpha^{\left(10-B\right)}$, where α is the smoothing factor set to 0.96 and B is the block number. This results in a weighting of 0.6925 for block 1 increasing exponentially up to 1 for block 10. For each variable, the value for each block is multiplied by the corresponding weighting then the average is taken to create the overall score for that variable. This score accounts for the potential change in performance over time and rewards those who are able to complete more of the assessment.

*Endurance and Activity Tolerance*: Heartrate is monitored throughout the assessment and is used to assess cardiovascular fitness. We calculated the average and max heartrate during each block. Additionally, after the completion of the assessment we monitored their heartrate for 3 min. The average for each minute is used to evaluate the participants ability to recover from exertion. At the end of each block, we record pain and rating of perceived exertion. Rating of perceived exertion was captured using the Borg Scale where the participant rated how hard they were working between 6 and 20 (26). Pain was reported on a 0 to 10 Numeric Pain Rating Scale for each location that they felt pain. The values and progression over time can indicate aspects of activity tolerance. The total distance completed is also used as a measure of endurance and activity tolerance.

*Gait Stability*: While the participant is walking at 1.6 m/s over the variable terrain, we record the position of markers on the feet and lumbar spine. From these markers we calculate temporal/spatial gait parameters as well as local dynamic stability (27, 28) to evaluate walking function and stability. The gait parameters included step length, step width, and step time. We calculated the average and standard deviation for each block. Local dynamic stability is based on dynamic systems theory and quantifies the ability of a system to maintain a stable cycle and recover from perturbations. It has been applied to the cyclical nature of human locomotion to quantify gait stability (27, 29) and has been shown to correlate with fall risk in multiple fall-prone populations (30–32). Local dynamic stability is calculated as the local divergence exponent of the lumbar spine marker with a greater number indicating greater instability.

*Identification and Recall*: The identification and recall task requires both the ability to scan the terrain and identify symbols, as well as remember them over the 400 m block (∼4 min) and after the completion of a weapon-based task. We calculated the number of errors that were made as the difference between number of each symbol reported and the number that were actually displayed. Both under-reporting and over-reporting were counted as errors. The error rate was calculated as the number or errors divided by the number of symbols presented. The error rate of the response is used to evaluate their recall ability.

*Motor Control*: During the weapon tracking task, participants must maintain a steady aim on a target while walking which requires control and coordination of the upper and lower body. We tracked the amount of time that the crosshairs were on the target. The percentage of time the crosshairs are on the target while the target is visible is used to quantify their ability to control their aim while moving. This was calculated for each of the 5 target presentations.

*Reaction Time and Decision Making*: The ambush task requires rapid, accurate decision making. We measure reaction time in milliseconds as the time between targets showing and being shot. Decision making was evaluated using the accuracy and precision of the shooting decisions. Accuracy was calculated as the percentage of correct responses out of the total number of responses. Precision was calculated as the percentage of the shoot targets that were shot. All of these measures are reported for single and double target presentations as well as overall for all targets.

### 3.3 Data processing and report generation

**Figure 5:**
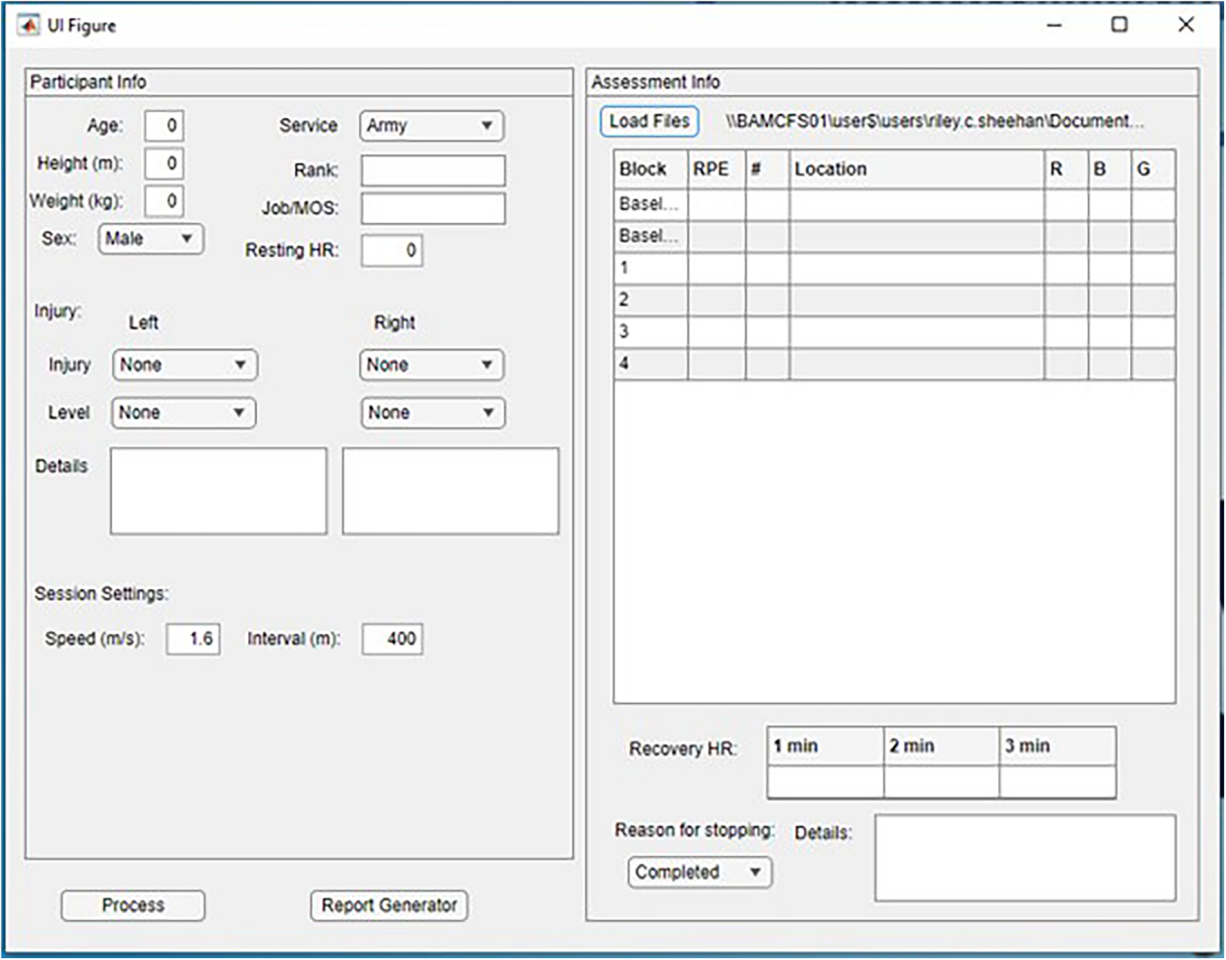
A screenshot of the data processing user interface. The interface allows for the entry of the data captured on the paper form as well as loading in the files collected during the assessment.

To maximize clinical utility, the data collection and processing pipeline was intentionally streamlined for simplicity and speed. Besides basic participant and session information that is recorded on a paper form, all data is captured in the same program. A custom graphical user interface was created to simplify the data entry and processing (Figure 5). To process the session, the data from the paper form is entered into the program and the output files are selected. The custom program reads in the files, calculates all relevant variables, saves them to a database, and generates a report of the session (Supplementary Material—Sample Report). The report can then be shared with the patient and clinician to inform discussions and treatment planning. From the end of the assessment, the data can be processed and a report generated in under 10 min.

### 3.4 Additional features

Care was taken to minimize the training and expertise needed to administer the assessment. The marker set was designed to have the minimal number of markers while still providing robust tracking. The use of an integrated virtual reality system also simplified the administration of the assessment. The entire assessment process can be completed with 2 personnel, a CAREN operator to run the scenario and a member of the clinical team to attach the sensors and collect session data. Additionally, the scenario was deliberately designed to maximize flexibility and usability. By consolidating the data collection into one system, it is straightforward to update the existing heartrate sensor or integrate additional sensors if needed. Each of the tasks and parameters can be turned on and off or adjusted to allow for use as part of a rehabilitation program or as an alternative assessment. While the use of REDOp2 is limited to facilities with a CAREN system, this flexibility increases the potential use-cases and utility of the scenario.

## 4 Results

One of the primary goals of the development of REDOp2 was to address the limitations identified in the original REDOp assessment (Table 1). This paper outlines those issues, how they were addressed, and the steps needed to validate the assessment.

The original assessment provided limited challenge and assessment of the cognitive domain. This was due to both only containing one cognitive task and that task not providing sufficient cognitive challenge. In REDOp2, we are able to evaluate identification and recall using the map symbol task and rapid decision making with the updated ambush shooting task. The updated ambush shooting task improves upon the original assessment by having a more complex rule set for determining friend or foe targets and requiring rapid decision making due to the targets only being visible for 3 s before disappearing. The combination of these tasks provides a much fuller assessment of cognitive function in a military-relevant environment.

Based on observations of patients completing the original REDOp assessment, it was noted that individuals were often rigid in their movements. This resulted in difficulty decoupling their upper and lower body movements and the inability to maintain a stable aim. The target tracking task in REDOp2 was designed to evaluate and quantify this aspect. By assessing participants in 5 different target presentation, the assessment is able to identify specific limitations that can be addressed through rehabilitation and training.

In the original REDOp, the movement of the treadmill was decoupled from the virtual environment and progressively got faster and steeper. This incongruence and lack of realism was a common complaint from participants. In REDOp2, we tied the treadmill and platform movement to the virtual terrain and maintained the speeds and distances within challenging, but military-relevant ranges. Specifically, the 4 km patrol, 1.6 m/s forced march pace, and 3–5 s rushes were drawn directly from profiles and training documents. This provided a more realistic and immersive experience while still providing a challenging assessment.

Due to the construction of the REDOp assessment around repeating blocks and participants completing as much as they can, it was difficult to compare the performance of participants that completed a different number of blocks. For example, it is hard to compare the high accuracy of someone who only completed 1 block with the decreasing accuracy of someone who completed 10 blocks. In REDOp we only focused on the performance during the first block. While this provided a common timepoint for comparison, it discounted the performance on the other blocks. For REDOp2, we utilized exponential weighted averages to create an overall session score for each variable. Because later blocks are more heavily weighted, performance decrements in later blocks are not penalized. While the specific weightings need to be tuned with further research, this approach allows for effective comparison of participants across assessments.

Lastly, the original REDOp required extensive setup and processing time from trained personnel. This limited utility and delayed reporting to SMs after their assessment. To address this, REDOp2 was developed to be as streamlined and automated as possible to reduce the time and training needed for setup and processing. The marker set was reduced from 57 to only 3 markers placed on the heels and the lumbar spine, which is manageable by any clinician. All data capture was consolidated so it was contained in a single file, removing the need to compile data from multiple programs and computers. Finally, a companion data processing program was developed to simplify the data entry and report generation process. In the end, REDOp2 is able to be conducted by 2 personnel with minimal setup and have a report generated for the patient within 10 min of completing the assessment.

In addition to improving on the original REDOp, the use of virtual reality as the basis for the assessment allowed us to address many of the limitations of current assessments. Based on comments and observations, the immersiveness of the scenario appears to elicit natural motions and responses from participants. Additionally, the flexibility of virtual reality allowed us to challenge and evaluate multiple domains, individually and simultaneously, all within a military-relevant context.

The REDOp2 assessment and the associated reports have been observed and reviewed by key stakeholders including members of the Military Physical Evaluation Board. The members of the Physical Evaluation Board, which is one of the primary groups that makes the official return to duty decision, were particularly excited about the potential of the scenario to bring much needed multidimensional, objective information about a service member's ability to perform military duties. Four representatives of the Physical Evaluation Board completed a survey where they rated on a 7-point Likert scale (1 = Low, 7 = High) the military relevance, ability to assess military demands, and potential to inform return to duty decision making for the different assessment components. All components and the overall assessment were rated as a 5 or greater with an average score of 6.4. The overall positive response from the stakeholders establishes the face validity of the assessment to evaluate military readiness.

While the REDOp2 assessment has promise, there are still some limitations that may restrict its adoption. The primary limitation is that the assessment is based on the CAREN system, which drastically reduces the locations where the assessment can be administered. There are a number of CAREN systems within the DoD across the country including the other Advanced Rehabilitation Centers at Walter Reed National Military Medical Center in Bethesda, Maryland and the Naval Health Research Center in San Diego, California. This level of assessment is likely not necessary for all injuries and military occupations. The REDOp2 assessment is most appropriate for highly trained and experienced wounded service members that require thorough assessment prior to returning to duty to ensure the safety of the team and mission. Due to the much smaller, but still critical, population that this would apply to, it would be feasible to assess them at one of the DoD CAREN sites.

## 5 Discussion

Our team has addressed the limitations of the original REDOp assessment to create a multidimensional military assessment, REDOp2. The feedback from the demonstration for the Physical Evaluation Board has established the initial face validity of the assessment. However, there are still a number of steps that need to be taken before REDOp2 is ready to be implemented to begin informing return to duty decision making. These steps include establishing reliability and validity, creating a normative database, and developing a comprehensive readiness score.

Test-retest reliability can be established by testing the assessment performance on multiple days. This will be critical if the assessment is to be used to track progress towards the goal of returning to duty. Additionally, the data captured by the embedded performance measures needs to be compared to existing, validated measures to establish concurrent validity. For this, each measure would be paired with existing measures, such as comparing the REDOp2 reaction time measure to the reaction time from the Defense Automated Neurobehavioral Assessment (33–35). This will ensure that REDOp2 is effectively measuring the intended domains. Through the testing of injured and healthy SMs, we will be able to build up a normative database. This will be invaluable for understanding how an injured SM's performance relates to the performance of their uninjured peers. Lastly, to simplify the interpretation of the data, a comprehensive readiness score needs to be developed. There is no gold standard measure of military performance, but methods like structural equation modeling have the potential to combine all of the individual measures from the assessment into a single index score that quantifies their overall readiness. The development and validation of this score would require extensive research, but would greatly improve the ability of the assessment to inform return to duty decision. Additionally, this readiness score could serve as the criterion score to base the development and validation of other less resource intensive, deployment-friendly assessments.

The team has taken the first step with the development of the REDOp2 assessment. By following the outlined steps, we will be able to set up the assessment for implementation. In the end, the goal is to have a reliable assessment of military performance that can help inform rehabilitation and return to duty decision making. Ultimately, this will help maintain and improve readiness.

## Data Availability

The original contributions presented in the study are included in the article/Supplementary Material, further inquiries can be directed to the corresponding author.

## Supplementary material

Supplementary Video 1 Ambush task video.

Supplementary Video 2 Tracking task video.

Supplementary Datasheet 1 Sample REDOp2 report.
