# Supplementary material for: Development of a multidimensional military readiness assessment
Source: Front Rehabil Sci. 2024 Mar 20;5:1345505. doi: 10.3389/fresc.2024.1345505 (PMC10987742; doi:10.3389/fresc.2024.1345505)

REDOp2 Report

Session: 221027_0900

Patient Info

Age: 36
Army, COL, 38W
Height: 1.30 m
Weight: 75.00 kg

Injury:
Left: Amputation, Below Knee, Details
Right: None, None,

Heart Rate:
Resting: 60 bpm
Max: 184 bpm

Session Settings

Standard
Speed: 1.60 m/s
Interval: 400 m
Sprint
Tracking
Ambush
Recon

Session Summary

Distance: 3003.2 m
Time: 15:13
Outcome: Pain, ouch
RPE: 15
Pain: 3 l foot, 1 back

Heart Rate:
Recovery: 90, 80, 60 bpm
Avg: 109 bpm
Max: 151 bpm

Scores:
Tracking: 73.4%
Recon: 0.4 error rate
Accuracy: 73.2%
Precision: 73.2%
Reaction Time: 1.23 s

Gait Measures:
Step Width: 7.5 (6.6) cm
Step Length: 0.7 (0.1) m
Step Time: 0.63 (0.14) s
Dynamic Stability: 0.50

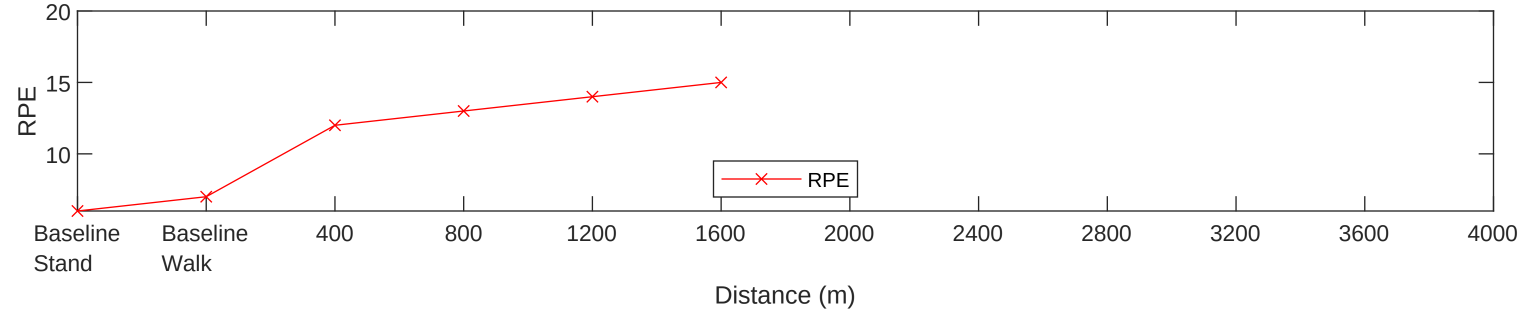

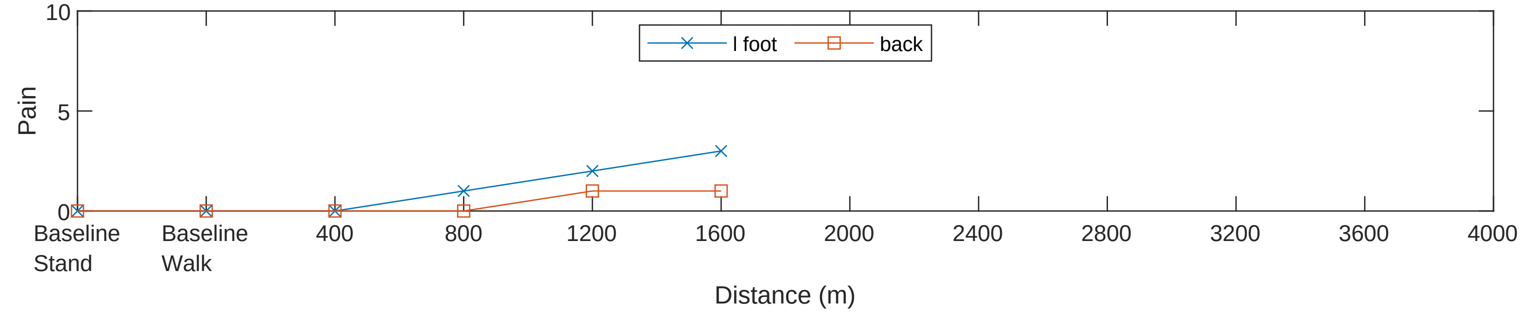

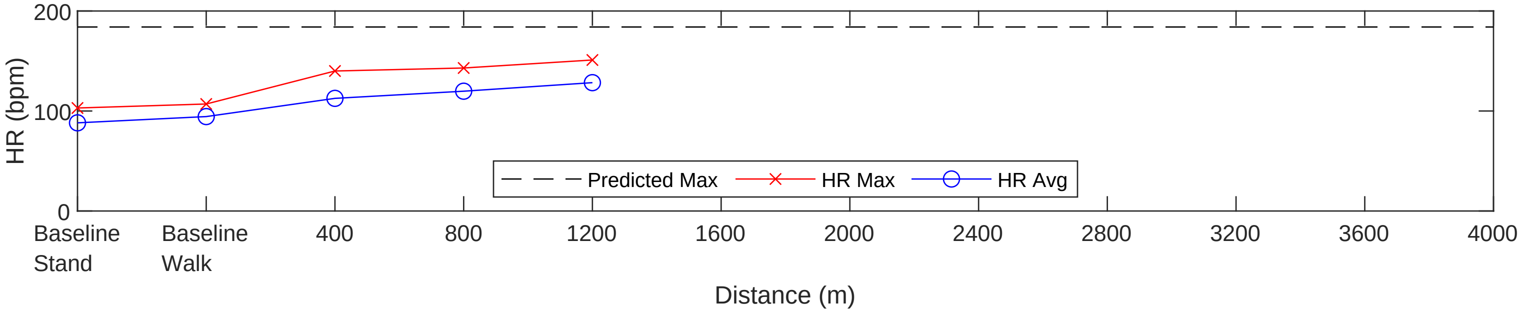

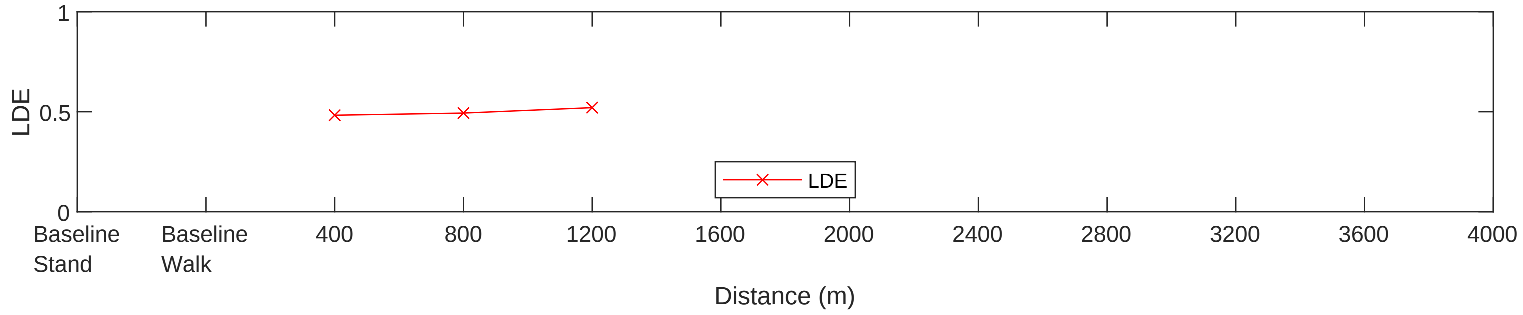

REDOp2 Report

Session: 221027_0900

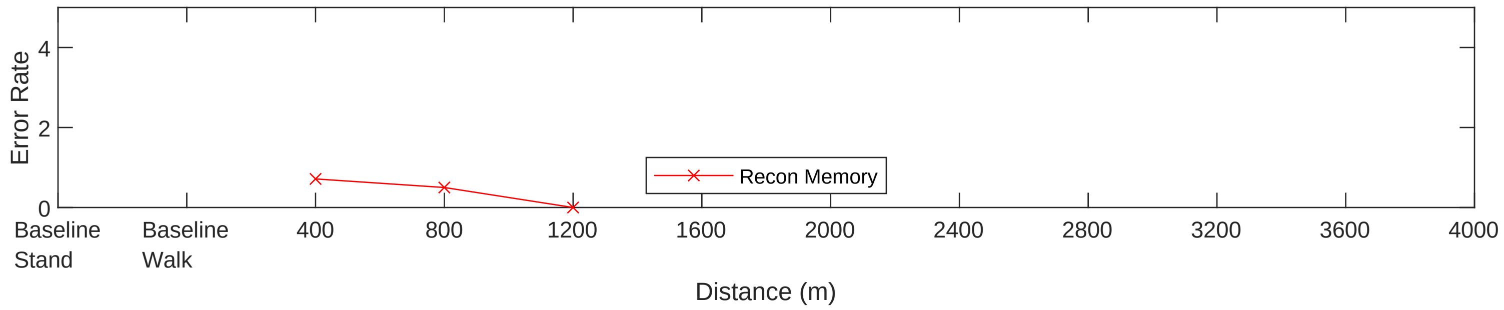

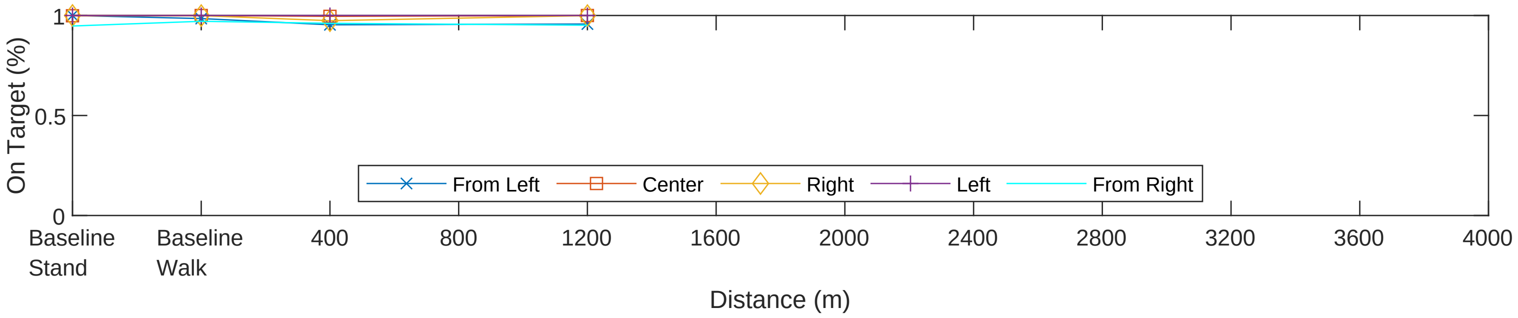

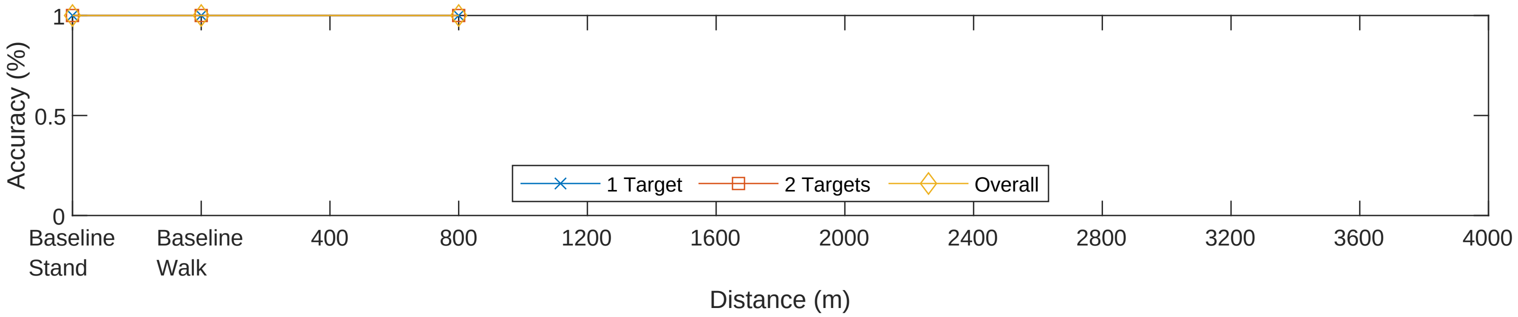

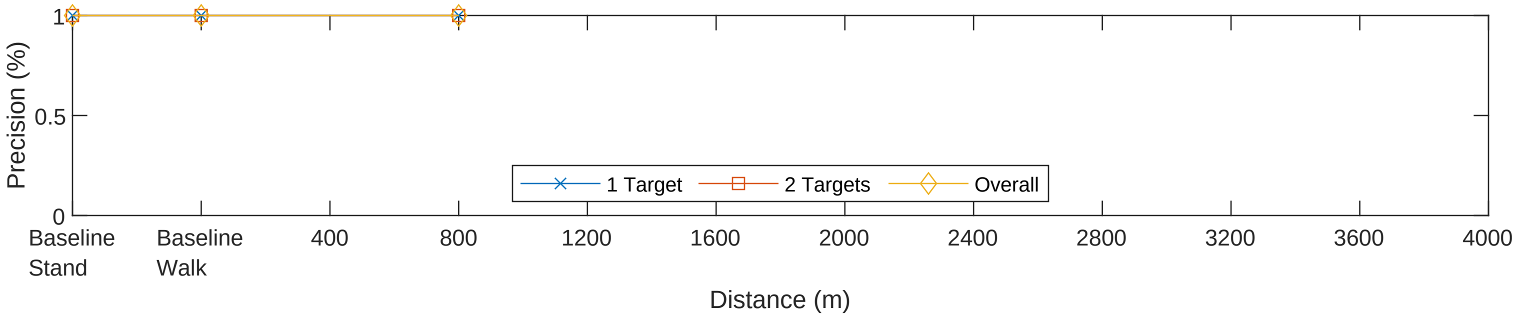

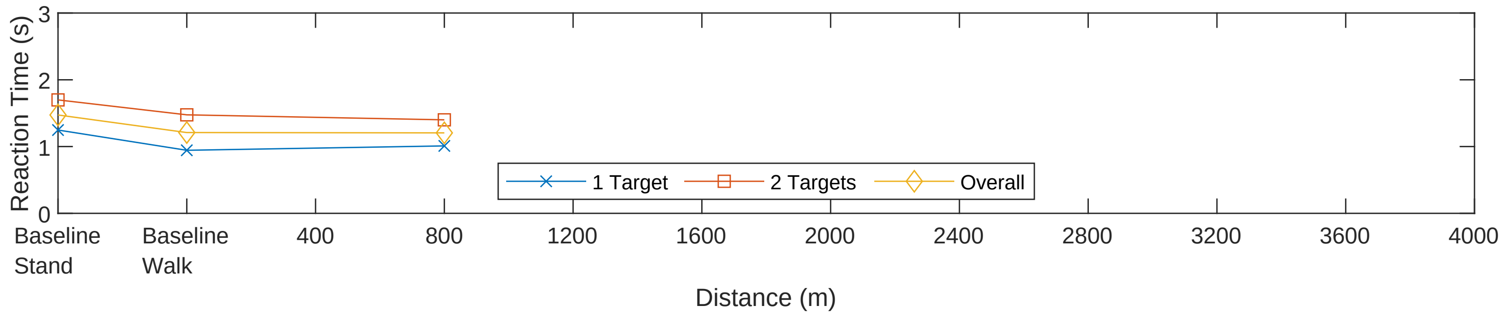

Supplement: Supplementary Datasheet 1 — Sample REDOp2 report. [file Datasheet1.pdf]
